# L-asparaginase-induced severe acute pancreatitis in an adult with extranodal natural killer/T-cell lymphoma, nasal type: A case report and review of the literature

**DOI:** 10.3892/ol.2014.1871

**Published:** 2014-02-11

**Authors:** FANG WU, LU QU, YAFEN TAN, YUE ZHANG, CHUNHONG HU

**Affiliations:** Department of Oncology, The Second Xiangya Hospital of Central South University, Changsha, Hunan 410011, P.R. China

**Keywords:** L-asparaginase-associated pancreatitis, adult, extranodal natural killer/T-cell lymphoma, nasal type

## Abstract

L-asparaginase (L-Asp)-associated pancreatitis (AAP) occurs occasionally; however, this side-effect has predominantly been observed among pediatric patients. Usually, it is not life-threatening and generally responds to intensive medical therapy. The present study presents a rare case of lethal AAP in an adult. The patient was recently diagnosed with extranodal natural killer/T-cell lymphoma (ENKTL), nasal type, and the chronic hepatitis B virus (HBV) infection and was receiving L-Asp as part of a chemotherapy regimen. Severe acute pancreatitis occurred and the patient succumbed 72 h after completion of chemotherapy. The HBV infection and lipid disorders may have been potential risk factors for the development of severe acute pancreatitis in the patient.

## Introduction

Extranodal natural killer/T-cell lymphoma (ENKTL), nasal type is associated with a poor prognosis and the outcomes of chemotherapy are considered to be unsatisfactory. Previous studies have reported that L-asparaginase (L-Asp)-based regimens may be an effective option to improve the chemotherapeutic efficacy on ENKTL (1–3). Acute pancreatitis is one of the predominant toxicities that is associated with L-Asp therapy (4). In the present study, a case of lethal L-Asp-associated pancreatitis (AAP) in an adult patient with ENKTL is presented, to raise awareness regarding the severe side-effects of L-Asp and the potential risk factors for developing lethal AAP in adults. Written informed consent was obtained from the patient’s family. The study was approved by the ethics committee of The Second Xiangya Hospital of Central South University (Changsha, China).

## Case report

**Figure 1:**
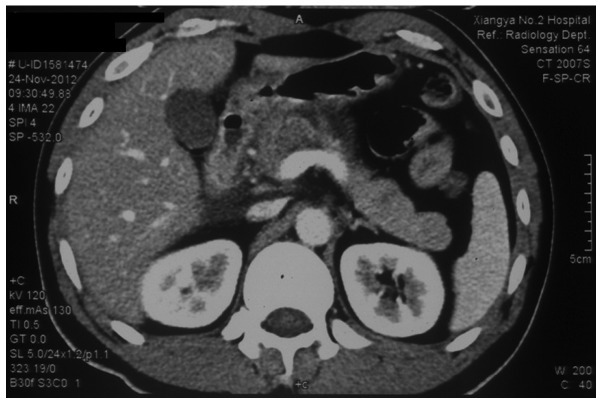
Computed tomography scan demonstrating diffuse swelling of the pancreas and peripancreatic fluid exudation.

A 29-year-old Chinese male was admitted to The Second Xiangya Hospital of Central South University (Changsha, China) due to ENKTL in October 2012. The patient’s medical history included the chronic hepatitis B virus (HBV) infection and a right front-parietal lobe transitional meningioma resection, which was conducted in June 2010. A physical examination was performed at the time of admission and the patient exhibited the following: Height, 173 cm; weight, 72 kg; and body surface area, 1.86 m^2^. The results of the biochemical analysis were within the normal limits with the exception of increased HBV-DNA copies (3.61×10^7^ IU/ml). In addition, an abdominal contrast-enhanced computed tomography (CT) scan showed no evidence of an abnormal pancreas, spleen or kidneys, however, fatty liver was observed. Combination chemotherapy was performed to treat the patient, which included intravenous injections of L-Asp (6000 IU/m^2^) for seven continuous days, 1000 mg/m^2^ gemcitabine on days one and eight and 85 mg/m^2^ oxaliplatin on day one. Additionally, the patient was administered with 0.1 g lamivudine per day as an antiviral treatment. Acute and severe epigastric pain occurred two days after completion of the chemotherapy and a physical examination revealed epigastric tenderness. The values of total bilirubin (44.4 μmol/l) and direct bilirubin (9.7 μmol/l) increased, serum amylase (964.2 μl), urinary amylase (219.7 μl) and fasting glucose (8.11 mmol/l) were significantly increased and the level of serum calcium (1.86 mmol/l) was markedly decreased, which indicated severe inflammation and dysfunction of the pancreas. The patient immediately received resuscitation fluid intravenously and a supplement of electrolytes. Meperidine and somatostatin were administered to relieve the pain, and to decrease the secretion of pancreatic fluid and total parenteral nutrition, respectively. A contrast-enhanced CT scan of the abdomen (Fig. 1) revealed diffuse swelling of the pancreas and peripancreatic fluid exudation, demonstrating acute pancreatitis. The patient presented with dizziness and exacerbation of abdominal pain on the subsequent night. A biochemical analysis of the serum demonstrated serum amylase, fasting glucose, calcium and serum triglyceride levels of 177.3 μl, 10.45, 0.57 and 3.85 mmol/l, respectively, indicating exacerbation of the pancreatic injury. On the next morning, the patient was thirsty and experienced heart palpitations. A physical examination identified tension in the abdominal muscles, epigastric tenderness, rebounding pain and the disappearance of gurgling sounds. The electrocardiogram identified a significantly higher heart rate with an undetectable blood pressure. The patient succumbed 72 h after completion of the chemotherapy.

## Discussion

ENKTL is a type of natural killer cell lymphoma, which has a greater incidence in Asian countries compared with Western countries. ENKTL accounts for 5–10% of all lymphomas in the Chinese population and is often associated with a poor prognosis. Radiation therapy is widely administered for the treatment of ENKTL and the addition of chemotherapy is encouraged to reduce the risk of recurrence (1). However, the effectiveness of a traditional chemotherapy regimen remains limited and ENKTL requires a different therapeutic approach. Numerous studies have reported that certain novel chemotherapeutic drugs may increase the response rate for ENKTL, including L-Asp (1–3). It has been demonstrated that L-Asp, in combination with gemcitabine and oxaliplatin (also termed GELOX) followed by radiation therapy, may be an effective and feasible treatment strategy. The overall therapeutic response to L-Asp was ≤96.3%. Furthermore, the two-year overall and progression-free survival rates were ≤86% (1). These results were supported by other studies, which revealed a marked antitumor effect of L-Asp-containing regimens (2,3). AAP occasionally develops, however, this side-effect is not usually life-threatening and generally responds to intensive medical therapy (4). There was one case of mild AAP reported in an adult patient with ENKTL (5). In the present study, it was hypothesized that the lethal AAP that occurred in the patient may have been related to additional factors.

A previous study demonstrated that L-Asp may lead to temporary hypertriglyceridemia, which is closely associated with acute pancreatitis (6). A retrospective study evaluated the lipid levels of 65 infants and adolescents that were treated with L-Asp; 12 patients exhibited TG levels >400 mg/dl (7). Seah *et al* (8) reported a case of L-Asp-induced hypertriglyceridemia in an adult natural killer T-cell lymphoma patient. Although the pathogenesis of L-Asp-associated hypertriglyceridemia remains unclear, an association between acute pancreatitis and hypertriglyceridemia has long been recognized. Therefore, it is considered to be necessary to monitor triglyceride levels prior to and during L-Asp therapy. Furthermore, a low-fat diet is considered to reduce the risk of complications induced by L-Asp. In addition, plasmapheresis may be an effective method to treat L-Asp-associated hypertriglyceridemia (9). In the present case, fatty liver was identified in the patient via a CT scan, which indicated a potential lipid disorder prior to chemotherapy. During the treatment with L-Asp, the triglyceride levels increased and using conservative diet control failed to regulate the levels. Taken together, these factors may be a potential cause for the initiation of lethal pancreatitis.

HBV is one of the etiological factors of acute pancreatitis (10–12). In the majority of cases, hepatitis-induced acute pancreatitis is mild and recovers with conservative management (10). Analysis of the serum from the patient identified four positive parameters that were associated with the HBV infection, namely HBsAg(+), HBeAg(+), IgG anti-HBc(+) and HBV-DNA copies (3.61×10^7^ IU/ml), which indicated that HBV was active in the patient. Following L-Asp therapy, the levels of total and direct bilirubin increased and severe pancreatitis occurred. Therefore, although HBV may not be the predominant cause of acute pancreatitis in the present case, it was hypothesized that it may have exerted an additive effect with L-Asp during the onset of AAP. Hepatitis B is an important public health issue in China. It was reported that 9% of the Chinese population are chronically infected carriers of HBV (13); thus, greater attention should be paid to HBV carriers during chemotherapy using L-Asp. Furthermore, the timing of L-Asp administration in a HBV-infected patient undergoing antiviral therapy requires investigation.

Although L-Asp has been demonstrated to be an effective treatment for ENKTL patients, it may induce severe acute pancreatitis independently or synergistically. Therefore, greater attention should be paid to this severe side-effect and the potential risk factors, which are associated with developing lethal AAP in adults. In the present study, it was indicated that the management of lipid disorders and anti-HBV therapy should be performed on an individual basis prior to and during the administration of L-Asp in ENKTL patients.
